# Editorial: What does not kill you makes you stronger: Interactions between environmental stresses and microbial virulence

**DOI:** 10.3389/fmicb.2022.1127058

**Published:** 2023-01-09

**Authors:** Liang Wang, Ling-Jun Zhan, Michael J. Wise

**Affiliations:** ^1^Laboratory Medicine, Guangdong Provincial People's Hospital (Guangdong Academy of Medical Sciences), Southern Medical University, Guangzhou, Guangdong, China; ^2^Centre for Precision Health, School of Medical and Health Sciences, Edith Cowan University, Perth, WA, Australia; ^3^Beijing Key Laboratory for Animal Models of Emerging and Reemerging Infectious Diseases, Beijing, China; ^4^Institute of Laboratory Animal Sciences, Chinese Academy of Medical Sciences, Comparative Medicine Center, Peking Union Medical College, Beijing, China; ^5^Department of Computer Science and Software Engineering, The University of Western Australia, Perth, WA, Australia; ^6^The Marshall Centre for Infectious Diseases Research and Training, The University of Western Australia, Perth, WA, Australia

**Keywords:** environmental stress, microbial virulence, sit-and-wait hypothesis, durability, transmission

The intriguing sit-and-wait hypothesis predicts that “virulence should be positively correlated with durability in the external environment because high durability reduces the dependence of transmission on host mobility” (Walther and Ewald, 2004) (Figure 1). Since the hypothesis was first proposed in the late 20th century (Ewald, 1983), both theoretical and computational studies have been reporting the relationships between environmental durability and microbial virulence (Sundberg et al., 2014; Wang et al., 2017, 2018, 2019a,b, 2020; Rafaluk-Mohr, 2019; Li et al., 2021). For example, Sundberg et al. (2014) investigated how long-term starvation influences the virulence of the fish pathogen *Flavobacterium columnare*, which revealed that abiotic selection did not solely facilitate high virulence but diversified virulence of the environmentally transmitting bacterial pathogen. In contrast, Wang et al. (2017) applied computational methods to investigate the relationships between bacterial durability and virulence, which showed that “non-vector-borne pathogens with sit-and-wait potentials have higher number of virulence and durability genes compared with other bacterial groups”, providing theoretically genetic basis for the hypothesis. However, debates have been revolving around the plausibility of the hypothesis due to the lack of direct molecular and genetic evidence (Turner et al., 2021; Pandey et al., 2022). Under this Research Topic, we sought to highlight an exciting set of groundbreaking efforts proposed by frontline investigators, which mainly focused on implementing studies to get an in-depth understanding of the relationships between environmental stress and bacterial virulence. Articles can be fitted into either of the five categories: (1) measures of virulence vs. abiotic stress tolerance; (2) application of sit-and-wait hypothesis to viral, fungal, archaeal or bacterial pathogens; (3) cross kingdom analyses; (4) patterns of abiotic stress tolerance; and (5) computational analysis of the links between abiotic stress tolerance and virulence.

**Figure 1:**
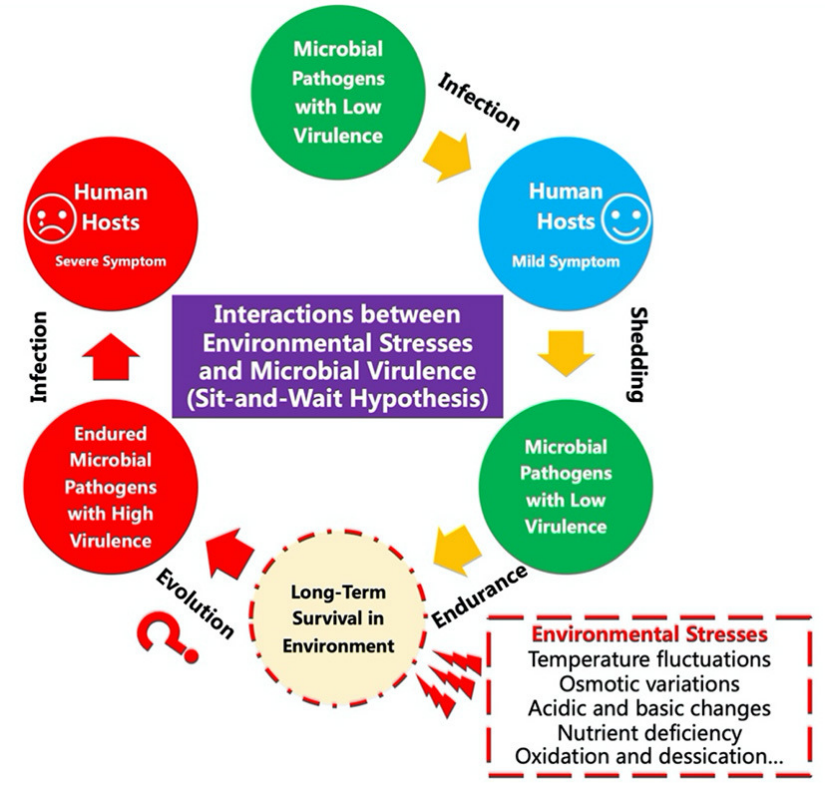
Schematic illustration of the sit-and-wait hypothesis in terms of the potential interactions between environmental stresses and the evolution of microbial virulence.

In particular, a total of four original research articles are enclosed in this Research Topic, which are either about bacterial responses to environmental stresses or about bacterial virulence factors and phenotypes. For example, Liu et al. studied the microevolution of the laboratory strains of *Pseudomonas aeruginosa* PAO1 attributed to selective media culture or prolonged passage in the laboratory by focusing on two genes, *mexT* and *lasR*, which are the two key components of the quorum sensing (QS) circuit. Through introducing these mutations into *P. aeruginosa*, it was found that they were related with QS, virulence, motility, and biofilm formation, which indicated that stress-induced microevolution could be correlated with bacterial virulence phenotypes, providing genetic basis for the sit-and-wait hypothesis. In the study of the impacts of proton irradiation on gut microbiota and its metabolome characteristics, Li et al. explored the changes of intestinal flora before and after proton irradiation stress by comparing the feces of Balb/c and C57BL/6J mice, which revealed that intestinal microbiome and some metabolites were related to the repair of intestinal injury. Therefore, the influences of the proton irradiation stress on complex systems like gut microbiota are more complex and difficult to establish the relationships between environmental stress and microbial virulence, which requires further investigations. In another *P. aeruginosa* study by Dela Ahator et al., virulence factor regulator (Vfr) controlling CRISPR-Cas system in response to abiotic stresses e.g., calcium deficiency was identified, which not only facilitated the design of effective phage therapies against *P. aeruginosa* but also revealed the presence of a regulatory network between virulence and abiotic stress in the bacterial pathogen *P. aeruginosa*. Lastly, Kuhl et al. sequenced and analyzed a plant-associated bacterium *Rhodococcus qingshengii* RL1, which revealed its stress tolerance features through the identification of a repertoire of genes enabling it to survive under different abiotic stress conditions. Although the study only revealed the benefits of these stress tolerance during plant-microbe interactions, it established methods for studying microbial stress tolerance and its related genes (Kuhl et al.), which could provide methodological references for studies focusing on the interactions between environmental stress and microbial virulence.

Taken together, through the studies enclosed in this Research Topic, recent progresses in the field of interactions between environmental stress and microbial virulence have been discussed, which provides insights into their complex relationships and facilitates our understanding of the sit-and-wait hypothesis. However, the relationship between environmental stresses and microbial virulence are still under intensive investigations for specific molecular mechanisms due to their extreme complexity on evolutionary time-scales, which will provide better guidance toward management and prevention of infectious diseases in future.

## Author contributions

LW drafted the manuscript. LW, L-JZ, and MW revised the draft. LW and MW made substantial contributions to the work through in-depth discussion. LW is a bioinformatician and microbiologist who leads the Theoretical and Experimental Microbiology (TEM) Group as a principal investigator at Laboratory Medicine, Guangdong Provincial People's Hospital (Guangdong Academy of Medical Science), Southern Medical University, Guangdong Province, China. LW is also an adjunct associate professor at Edith Cowan University, Perth, Western Australia, Australia. All authors proposed the Research Topic theme, made a direct and intellectual contribution to the work, and approved the final version of the editorial for publication.
